# Disrupted sleep-wake cycles and circadian rhythms in a *Drosophila* model of *C9orf72*-FTD

**DOI:** 10.3389/fnins.2026.1814072

**Published:** 2026-05-13

**Authors:** Kendall E. Eby, Braeden R. Shields, Isabella DelNegro, Sarah Morley, Pamela A. Snodgrass-Belt, Marla Tipping

**Affiliations:** Department of Biology, Providence College, Providence, RI, United States

**Keywords:** activity level changes, *C9orf72*, circadian rhythm, Frontotemporal dementia FTD (FTD), neurodegeneration, sleep depth, sleep disruption, sleep quality

## Abstract

Frontotemporal dementia (FTD) is a neurodegenerative disorder that affects behavior, personality, motor activity, speech, cognition, and sleeping patterns. Previous findings support the idea that disruption of sleep and circadian systems may not only be affected by this disease but also work to actively shape the clinical phenotype of FTD. Thus, understanding how sleep-wake cycles are altered may provide insight into mechanisms that influence both disease progression and quality of life. We studied an established *Drosophila* model of FTD to investigate changes in the sleep-wake cycle of both young and aging flies. A *C9orf72-*associated FTD model was chosen, as the most common genetic cause of sporadic and hereditary FTD is a hexanucleotide repeat expansion in intron 1 of the *C9orf72* gene. We performed behavioral assays to measure locomotor activity in both a 12 h:12 h light/dark (LD) cycle and complete darkness (free running). From this data, we were able to analyze changes in sleep and activity patterns, as well as circadian rhythms in flies modeling *C9orf72*-FTD. Our data suggests that these flies have increased nighttime activity and decreased sleep at night, which becomes more significant as they age. Older flies also displayed decreased sleep pressure during both day and night and lost rhythmicity. Of specific interest, young flies modeling *C9orf72*-FTD demonstrated altered day and night sleep latency, decreased sleep depth at night, and reduced rhythmicity in constant darkness. This suggests that changes in their sleep-wake cycle occur early in disease progression and provide an avenue for potential intervention and early diagnostic markers.

## 1 Introduction

Frontotemporal dementia (FTD) is a neurodegenerative disorder that affects behavior, personality, motor activity, speech, cognition, and sleeping patterns. The predominant genetic cause of FTD is a GGGGCC (G4C2) hexanucleotide repeat expansion in intron 1 of the *C9orf72* gene. This expansion drives neurodegeneration through a combination of haploinsufficiency, toxic repeat RNA, and dipeptide repeat protein (DPR) accumulation (DeJesus-Hernandez et al., 2011; Renton et al., 2011). Specifically, abundant DPR inclusion has been detected in human brain tissue from post-mortem patients with ALS and/or FTLD-TDP with *C9orf72* repeat expansion (Dedeene et al., 2019). Patients with *C9orf72*-FTD have also been found to have increased disruptions in circadian rhythmicity and rest-activity cycles, including reduced morning activity, decreased total sleep, and impaired sleep efficiency. This indicates that clock-regulated behavior is strongly affected in this disease (Anderson et al., 2009; Walker and Vaughn, 2021; Larsen et al., 2023). Multiple studies in *Drosophila*, rodent models and human post-mortem tissue link clock gene dysregulation to neurodegeneration (Namgyal and Lim, 2025). This suggests that circadian dysfunction may contribute mechanistically to disease expression rather than arising solely due to neurodegeneration. Together, these findings support the idea that disruption of sleep and circadian systems may actively shape the clinical phenotype of FTD.

Sleep quantity and quality are fundamental determinants of brain and body health. A reduction in either sleep duration or sleep quality is associated with both adverse mental and physical health outcomes, which highlights the importance of understanding both how well and how long an organism sleeps (Wiggin et al., 2020). Sleep is governed by a homeostatic balance between sleep and wake that is shaped by internal states and external environments, including diet, stress, and social conditions (Dubowy and Sehgal, 2017). Sleep goes beyond a state of passive downtime and is an actively regulated biological process reflective of neural and physiological processes. Sleep quality can be organized into two independent components—sleep pressure, quantified by the probability of entering sleep [P(Doze)], and sleep depth, quantified by the probability of awakening [P(Wake)]—which are altered by both internal and external factors such as dopamine, diet, aging, and sleep deprivation regardless of total sleep time (Wiggin et al., 2020). Aging-specific alterations reveal disrupted sleep structures that are likely relevant to age-associated neurodegeneration (Wiggin et al., 2020).

Circadian rhythm controls the sleep-wake cycle and thus influences sleep quality. Circadian clocks coordinate hormonal, metabolic, and behavioral rhythms across tissues, playing a central role in maintaining physiological homeostasis; disruption of this system may lead to wide-ranging consequences for health and disease risk (Tataroglu and Emery, 2014). At the cellular level in *Drosophila*, circadian timing is generated by a conserved feedback loop in which the genes *dClock* (*Drosophila CLK*), ortholog of mammalian *Clock*, and *Cycle* (*CYC*), ortholog of mammalian *BMAL1*, drive expression of *period* (*per*) and *timeless* (*tim)*, whose protein products work to inhibit their own transcription. In *Drosophila*, this machinery is embedded within a network of approximately 150 clock neurons that convert this intracellular cycling into coordinated rhythmic behavior outputs, similar to the mammalian suprachiasmatic nucleus (SCN) (Dubowy and Sehgal, 2017). These rhythms are mainly entrained by the external factors of light and temperature cycles, with light serving as the major zeitgeber. These factors allow the clock to align internal physiology with the external world (Tataroglu and Emery, 2014).

Frontotemporal dementia (FTD) presents a particularly compelling context for studying sleep and circadian dysfunction. Due to the broad link between worsening physical and mental health with sleep disruption and circadian dysregulation, these abnormalities represent potentially modifiable factors that contribute to neurodegenerative disorders such as FTD (Wiggin et al., 2020; Dubowy and Sehgal, 2017). Understanding how sleep pressure, sleep depth, and circadian organization are altered in *C9orf72*-FTD may provide insight into mechanisms that influence both disease progression and quality of life. Although substantial evidence indicates that sleep quality and circadian rhythms are disrupted in *C9orf72*-FTD, they have not yet been systematically quantified in disease models. At present, no study has jointly examined sleep pressure, sleep depth, circadian structure, and rest-activity organization in *C9orf72*-FTD *Drosophila*. To further investigate this relationship, we performed behavioral assays using the Drosophila Activity Monitoring (DAM) System, which enables continuous measurement of locomotor activity in both a 12 h:12 h light/dark (LD) cycle and complete darkness (DD, free running). From this data, we were able to analyze changes in sleep and activity patterns, as well as circadian rhythm in flies modeling *C9orf72*-FTD. Sleep pressure and depth were also analyzed from these assays using P(Doze) and P(Wake) metrics (Wiggin et al., 2020).

## 2 Materials and methods

### 2.1 Fly stocks

Flies were reared on standard food containing black strap molasses (100 mL), agar (14.9 g), cornmeal (100 mL), Brewer's yeast (41.2 g), and fungicide (2.25 g Tegosept dissolved in 22.5 mL 95% ethanol and 8 mL propionic acid) in deionized water (1,800 mL), at 25 °C and 65% relative humidity under a 12 h:12 h LD cycle. The following lines were obtained from the Bloomington *Drosophila* Stock Center: *P{w*^+*MC*^ = *UAS-LDS-(G4C2)12.GR-GFP}2, w*^1118^ (#84722), *w*^1118^*; P{w*^+*mC*^ = *UAS-LDS-(G4C2)44.GR-GFP}9* (#84723), and *P{w*^+*mC*^ = *GAL4-elav.L}2/CyO* (#8765). Progeny from *P{w*^+*mC*^ = *UAS-LDS-(G4C2)12.GR-GFP}2, w*^1118^ (#84722) flies mated with *{w*^+*mC*^ = *GAL4-elav.L}2/CyO* (#8765) flies will be considered control, and progeny from *w*^1118^*; P{w*^+*mC*^ = *UAS-LDS-(G4C2)44.GR-GFP}9* (#84723) flies mated with *{w*^+*mC*^ = *GAL4-elav.L}2/CyO* (#8765) flies will be considered our pathogenic model (experimental).

### 2.2 Locomotor and circadian assays

Virgin female flies were collected from *P{w*^+*mC*^ = *UAS-LDS-(G4C2)12.GR-GFP}2, w*^1118^ *and w*^1118^*; P{w*^+*mC*^ = *UAS-LDS-(G4C2)44.GR-GFP}9* lines and mated with *P{w*^+*mC*^ = *GAL4-elav.L}2/CyO* males, respectively. Crosses were established and maintained under a 12 h:12 h LD cycle. Shortly after eclosion, male flies were collected for 0–2 days and aged for 3 days (3–5-day old) or 12 days (12–15-day old) in LD until the locomotor and circadian assays were performed. Interpretation of female fly behavioral data is inherently more complex because mating status can substantially influence locomotor activity, sleep, and circadian patterns in *Drosophila* (Akpoghiran et al., 2025). Long term locomotor recordings, described below, create an additional issue due to egg-laying and hatching of larvae, which may alter readings and result in high activity counts or low sleep counts. While specialized food can inhibit egg hatching, this adds an additional variable. Due to these concerns, we analyzed only male flies to increase uniformity and avoid egg-laying artifacts.

We recorded all locomotor activity using the *Drosophila* Activity Monitoring (DAM5H) System (Trikinetics) over an average of 5 days in LD and 7 days in DD (constant dark). Flies were individually placed in small glass capillary tubes (5 mm diameter) containing standard food and a cotton plug. Control progeny (*UAS-LDS-(G4C2)12, w*^1118^*/y; elav-GAL4/*+) and pathogenic progeny (*w*^1118^*/y; elav-GAL4/*+*; UAS-LDS-(G4C2)44/*+) were tracked on the same monitor in groups of 16 (each monitor holds 32 tubes). This was repeated four times with progeny from independent crosses. The DAM5H activity monitor contains 15 independent infrared beams that detect activity. Activity was measured as the number of beam crosses (total counts) recorded at 1-min intervals. Flies were visually monitored each day to assess fly death. Flies that died prior to the end of the monitoring period were omitted from analysis.

### 2.3 Statistical analysis

The DAMFileScan program (TriKinetics) was used to create individual data files for each fly, binned in 1 min and 30 min increments. Sleep and activity were quantified in Matlab using SCAMP scripts (Vecsey et al., 2024). Locomotor activity was collected in 1 min and 30 min bins, and sleep was defined as five or more minutes of inactivity (Hendricks et al., 2000; Shaw et al., 2000). Sleep was analyzed by number of sleep episodes, total sleep duration, mean sleep episode duration, and sleep latency (time to first sleep episode following a light on or light off), averaged over 5 days. Sleep quality and depth were analyzed by calculating P(Doze) and P(Wake), respectively (Wiggin et al., 2020). Activity was analyzed by the number of activity episodes and total activity duration. Sleep and activity data were analyzed using a three-factor ANOVA with genotypes, age, and phase (light vs. dark) as factors. As day and night measurements were obtained from the same flies, light/dark was treated as a repeated measure. Main effects and all interaction terms were evaluated. There was no significant three-way interaction, but there were significant two-way interactions between age and genotype. Due to this, and because light and dark phases are biologically distinct and commonly analyzed separately in circadian and sleep studies, phase-specific analyses were also performed using two-way ANOVA with genotype and age as factors. These two-way ANOVA tests were followed by Tukey's multiple comparisons test. Statistical significance was defined as *p* < 0.05. Faas (Fly activity analysis suite software) developed by M. Boudinot and F. Rouyer (https://neuropsi.cnrs.fr/en/group-leader-francois-rouyer/) was used to analyze the average activity of 16 flies for each genotype at each time interval throughout an entrained circadian cycle (education). This was performed for 3–5-day-old and 12–15-day-old control flies, and 3–5-day-old and 12–15-day-old pathogenic flies.

Circadian rhythm was analyzed by ClockLab (Actimetrics), and Faas (https://neuropsi.cnrs.fr/en/groupleader-francois-rouyer/). In ClockLab, we performed a double-plot of the daily activity (actogram) of each single fly recording, using 10 min bins. Using Faas software, we analyzed rhythmicity, period, power of rhythm, robustness of rhythm, and morning anticipation in 3–5-day-old and 12–15-day-old control flies, and 3–5-day-old and 12–15-day-old pathogenic flies. Cycle_p (Faas software) was used to perform periodogram analysis of Sokolove and Bushell to find periodicity in fly activity in flies maintained under constant darkness (free-running) (Sokolove and Bushell, 1978). Rhythmicity was analyzed as a binary outcome (rhythmic vs. arrhythmic). Genotype differences were assessed within each age group using Fisher's exact test, and consistency of the genotype effect across age was evaluated using a stratified 2 × 2 analysis. Arrhythmic flies were removed from period analysis but were included in power of rhythm and rhythm robustness scores. Rhythm robustness is expressed as a percentage of the maximal Qp value (Qp = N for a perfect rhythmic activity). Education analysis in Faas was used to measure anticipation phase scores in flies maintained in 12 h:12 h LD. Anticipation phase score is a measure of the activity variation in advance of light-on (morning anticipation) and was defined as the percentage of activity in the 6 h period before lights-on that occurred in the 3 h just before the transition to lights-on (Harrisingh et al., 2007). Period, power of rhythm, rhythm robustness, and anticipation were analyzed for significance by two-way ANOVA tests for age and genotype factors, followed by Tukey's multiple comparisons test (or Šídák's multiple comparisons test for anticipation phase scores). Statistical significance was defined as *p* < 0.05. Prism 10 (GraphPad Software) was used for statistical analysis.

## 3 Results

### 3.1 Flies modeling *C9orf72*-FTD display altered activity patterns compared to control flies

To model *C9orf72*-FTD, we crossed flies carrying the expanded hexanucleotide repeat in intron 1 of the human *C9orf72* gene to *elav-GAL4* flies to drive neuronal expression. There have been many transgenic flies created that carry this expanded repeat. We chose to use the *UAS-LDS-G4C2 (44)* and *UAS-LDS-G4C2 (12)* lines generated by Goodman et al. (2019a), as they include a 114-nucleotide leader sequence (LDS) immediately upstream of the hexanucleotide repeat. This region is likely to influence RAN translation, which is responsible for the pathogenetic dipeptides produced from this repetitive sequence (Zu et al., 2018). The UAS-LDS-G4C2 (44) line contains up to 44 hexanucleotide repeats and has been studied to understand the molecular mechanisms of *C9orf72* pathogenicity (Goodman et al., 2019a,b). The UAS-LDS-G4C2 (12) line was generated as a control and carries up to 12 hexanucleotide repeats, which has been shown to result in no noticeable phenotypes nor detectable dipeptide production (Goodman et al., 2019a). In this study, we will refer to progeny expressing the UAS-LDS-G4C2 (44) construct as pathogenic, and progeny expressing the UAS-LDS-G4C2 (12) construct as control.

To study how progression of FTD affects sleep and activity, we utilized the *Drosophila* Activity Monitoring system (DAM5H) to investigate the locomotor behavior of male flies expressing a pathogenic number of hexanucleotide repeats in intron 1 of the human *C9orf72* gene compared to control flies. Flies over-expressing the pathogenic repeats have a reduced lifespan, dying by 30 days post-eclosion. This is consistent with other *Drosophila* models of *C9orf72*-FTD (Mizielinska et al., 2014) and in contrast to the 70-day median lifespan of healthy lab-maintained *Drosophila* (Ziehm et al., 2013). Due to this, we chose to monitor locomotor behavior in young flies (3–5-days old) and older flies (12–15-days old). Young flies were aged to at least 3 days old to allow adult sleep behavior to stabilize (Shaw et al., 2000), and flies were maintained and monitored in a 12h:12h light/dark (LD) cycle over a 5-day period.

**Figure 1:**
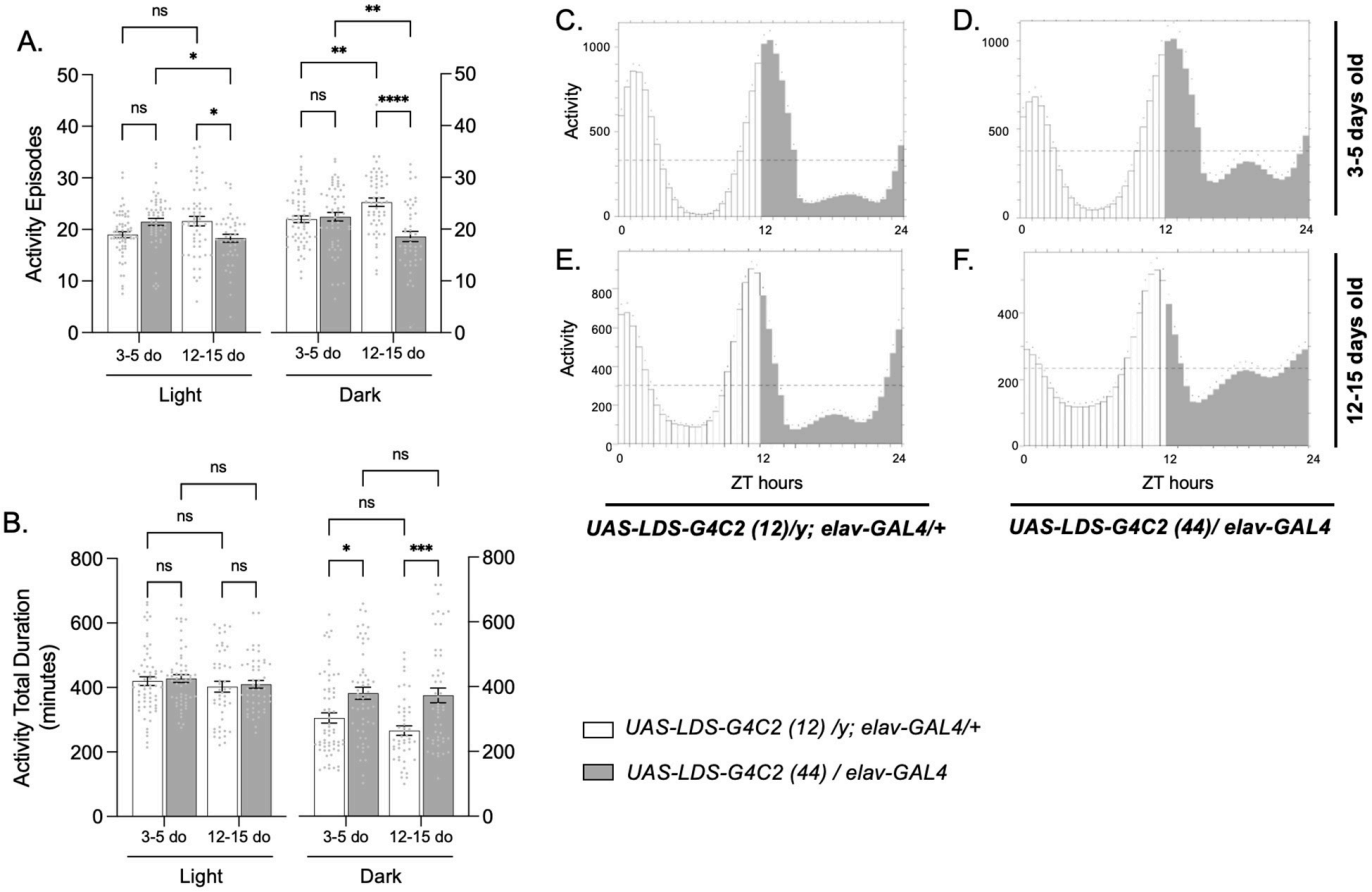
Flies modeling *C9orf72*-FTD display altered activity patterns compared to control flies. The average activity episodes **(A)**, and activity total duration **(B)**, of 3–5-day old and 12–15-day old *UAS-LDS-G4C2 (12)/y; elav-GAL4/*+ control flies (white bars) and *UAS-LDS-G4C2 (44)/ elav-GAL4* pathogenic flies (gray bars). The left side of each graph represents data obtained during the day (light), and the right side of each graph represents data obtained during the night (dark). Flies were monitored for an average of 5 total cycles. The mean, SEM, and all data points are shown. For young flies (3–5 do), *N* = 62 for control flies and 52 for pathogenic flies. For older flies (12–15 days old), *N* = 55 for control flies and 44 for pathogenic flies. *p < 0.05, **p < 0.01, *** *p* < 0.001, ****p < 0.0001, ns, not significant. Two-way ANOVA with genotype and age as factors, with light and dark analyzed separately, followed by Tukey's multiple comparisons test. Average daily locomotive activity rhythms (eduction) of *UAS-LDS-G4C2 (12)/y; elav-GAL4* /+ control **(C, E)** and *UAS-LDS-G4C2 (44)/elav-GAL4* pathogenic **(D, F)** flies at 3–5-days old **(C, D)**, and 12–15-days old **(E, F)**. ZT, Zeitgeber time, or hours since lights on. Mean is graphed as a dotted line, and SEM is represented by dots above bars. *N* = 16 for each genotype and age. Flies in **(A–F)** were monitored under a 12 h:12 h LD cycle.

Average activity episodes of young pathogenic flies were not significantly different from control flies during either the day (light) or night (dark) (Figure 1A, left); however, the average total duration of activity at night compared to controls was significantly increased (Figure 1B, left). As pathogenic flies aged, their average activity episodes were significantly reduced during the day and night in comparison to both their younger selves and same-aged control flies (Figure 1A, right), though the average total duration of activity at night was still significantly increased (Figure 1B, right). This suggests low-frequency, high-duration movement patterns at night, behavior that is often associated with older flies (Giebultowicz and Long, 2015).

To visualize the activity patterns of flies modeling *C9orf72*-FTD over the 12 h:12 h LD cycle, we analyzed average daily locomotive activity rhythms (eduction) of 3–5-day (top row) and 12–15-day (bottom row) old control (left) and pathogenic (right) flies. Control flies showed a reduced amplitude of activity as they aged (Figures 1C, E), but maintained a similar consolidated pattern, with the most activity occurring immediately after lights on and during the transition to lights off. However, even in young pathogenic flies, there was a noticeable increase in activity during the dark period (Figure 1D), and as they aged, a constant low level of activity throughout the day and night was observed (Figure 1F).

### 3.2 Flies modeling *C9orf72*-FTD display altered sleep patterns, sleep pressure and sleep depth

**Figure 2:**
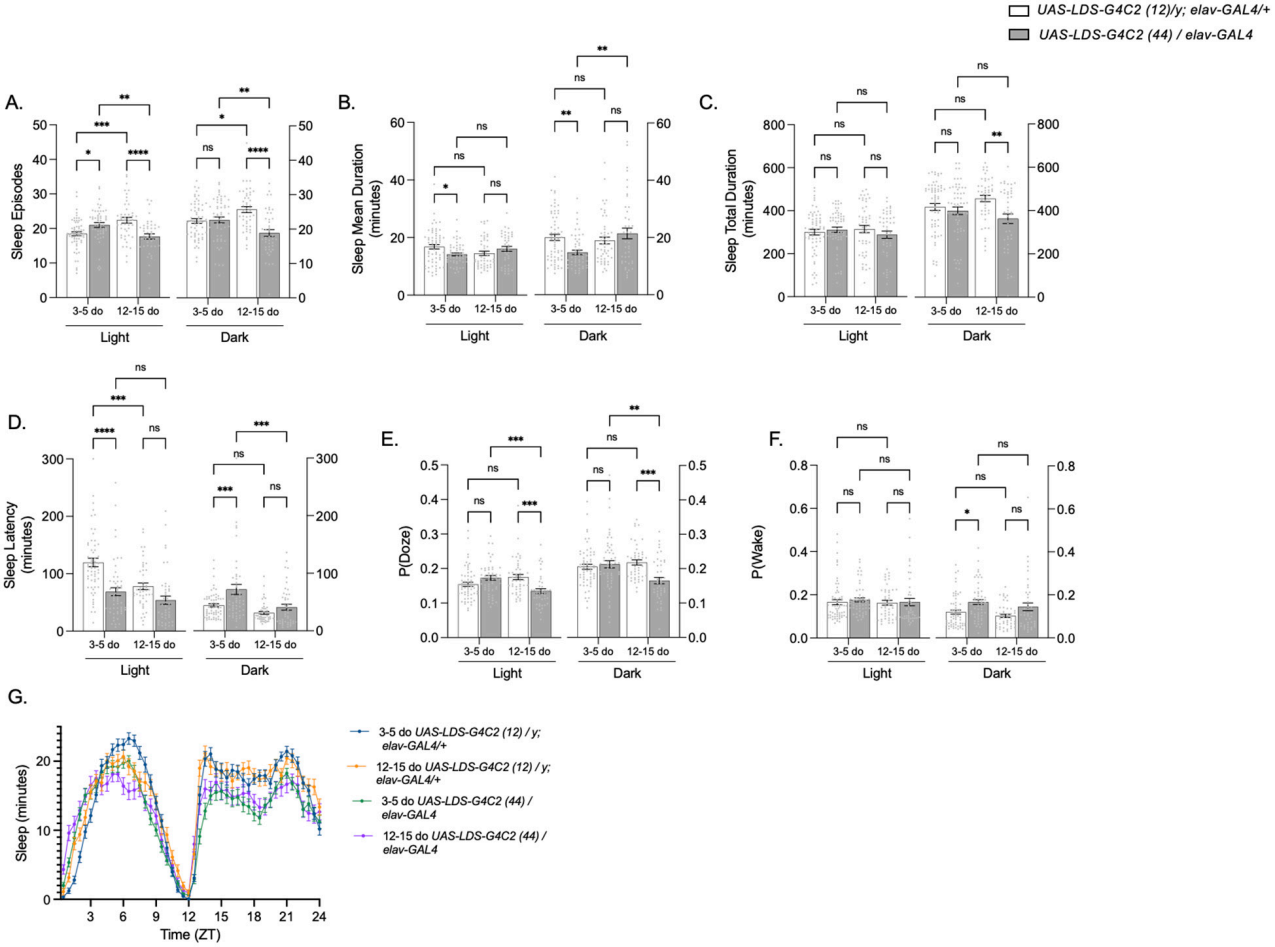
Flies modeling *C9orf72*-FTD display altered sleep patterns, increased sleep pressure during the day (light) and decreased sleep pressure at night (dark), as well as a decrease in sleep depth at night (dark) as they age. The sleep episodes **(A)**, sleep mean duration **(B)**, sleep total duration **(C)**, average sleep latency **(D)**, probability that an active fly will stop moving [P(Doze)] **(E)**, and probability that a stationary fly will start moving [P(Wake)] **(F)** of 3–5-day old (left) and 12–15-day old (right) *UAS-LDS-G4C2 (12)/y; elav-GAL4/*+ control flies (white bars) and *UAS-LDS-G4C2 (44)/elav-GAL4* pathogenic flies (gray bars). The left side of each graph represents data obtained during the day (light), and the right side of each graph represents data obtained during the night (dark). The mean, SEM, and all data points are shown. * *p* < 0.05, **** *p* < 0.0001, ns, not significant. Two-way ANOVA with genotype and age as factors, with light and dark analyzed separately, followed by Tukey's multiple comparisons test. Minutes of sleep per 30-min bin for each genotype and age **(G)** were graphed together for comparison. ZT, Zeitgeber time, or hours since lights on. Blue = 3–5-day old control, orange = 12–15-day old control, green = 3–5-day old pathogenic, purple = 12–15-day old pathogenic flies. The mean and SEM are shown. Flies in **(A–G)** were monitored under a 12 h:12 h LD cycle. Flies were monitored for an average of 5 total cycles. For young flies (3–5 days old), *N* = 62 for control flies and 52 for pathogenic flies. For older flies (12–15 days old), *N* = 55 for control flies and 44 for pathogenic flies.

Using the same data sets, we analyzed sleep patterns. Young pathogenic flies displayed a small but significant increase in sleep episodes during the day (Figure 2A, left). However, with a significantly reduced average mean duration of each sleep episode (Figure 2B, left), the total sleep duration for the 24 h cycle remained the same as that of their control counterpart (Figure 2C, left). In the dark, young pathogenic flies demonstrated a significantly shorter mean duration of sleep per sleep episode (Figure 2B, right), but total sleep duration was not changed (Figure 2C, right). As pathogenic flies aged, the average number of sleep episodes significantly decreased in both the light and dark periods (Figure 2A, left and right), resulting in a slightly decreased average total sleep duration in the light (though not significant) (Figure 1C, left) and a significantly decreased average total sleep duration in the dark (Figure 1C, right). Minutes of sleep per 30 min bin were graphed for all ages and genotypes in Figure 2G for direct comparison throughout the 24 h light/dark cycle. These observed reductions in sleep at night in flies modeling *C9orf72*-FTD, along with observed increases in activity, mirror flies modeling Alzheimer's Disease (Buhl et al., 2019), and Huntington's Disease (Farágo et al., 2019), as well as FTD patient reports of disrupted sleep-activity cycles and decreased total sleep (Anderson et al., 2009; Walker and Vaughn, 2021; Larsen et al., 2023).

To understand the mechanics of how sleep is affected in flies modeling *C9orf72*-FTD, we analyzed sleep latency. Sleep latency can be observed in the light period by measuring how many minutes it takes a fly to fall asleep after lights have turned on and can be observed in the dark period by measuring how many minutes it takes a fly to fall asleep after lights are turned off. This is interpreted as day sleep latency and night sleep latency, respectively. Interestingly, young pathogenic flies displayed altered day and night sleep latency, with flies falling asleep more quickly after lights are turned on (Figure 2D, left) and taking longer to fall asleep after lights are turned off (Figure 2D, right) compared to control flies. While a similar trend was observed with older pathogenic flies, there was no significant difference. Sleep latency is a common measure of sleep pressure (Linford et al., 2012); thus, we were interested in investigating the quality of sleep in *C9orf72*-FTD flies.

Sleep quality can be organized into two independent components – sleep pressure, quantified by the probability of entering sleep [P(Doze)], and sleep depth, quantified by the probability of awakening [P(Wake)] (Wiggin et al., 2020). Young pathogenic flies showed a decreased sleep latency in the light (Figure 2D, left), which indicates an increased sleep pressure, as the flies first sleep episode after lights turn on occurs more quickly than in control flies. We observed only a slight, non-significant, increase in P(Doze) (Figure 2E left) and no significant changes in sleep pressure in the dark (Figure 2E, right). However, as pathogenic flies aged, they exhibited a significantly reduced sleep pressure both in the light (Figure 2E, left) and dark (Figure 2E, right). Sleep depth, measured by P(Wake), was not significantly affected in the light period for young or older pathogenic flies (Figure 2F left). In the dark, young pathogenic flies displayed a lack of sleep depth indicated by an increase in the probability of awakening (Figure 2F, right). This same trend was observed in older pathogenic flies, but it was not significant. Collectively, we observed a decline in sleep quality as pathogenic flies age, and, intriguingly, a lack of sleep depth and measurable changes in sleep latency even in young pathogenic flies.

### 3.3 Flies modeling *C9orf72*-FTD exhibit advanced anticipation in LD and demonstrate reduced rhythmicity in constant darkness

**Figure 3:**
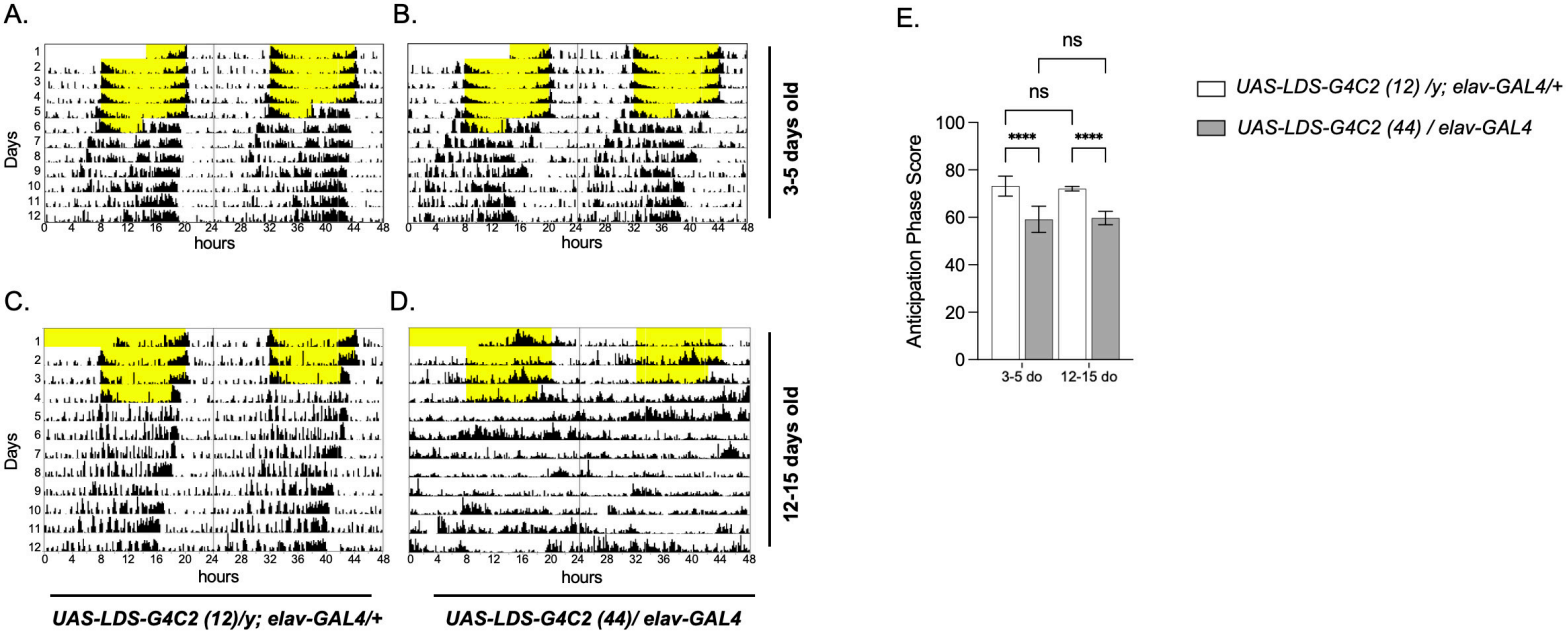
Young and older flies modeling *C9orf72*-FTD exhibit advanced morning anticipation in LD, and demonstrate reduced rhythmicity in constant darkness. Representative double-plotted activity recording (actogram) of a single fly recording from *UAS-LDS-G4C2 (12)/y; elav-GAL4/*+ control **(A, C)** and *UAS-LDS-G4C2 (44)/elav-GAL4* pathogenic **(B, D)** at 3–5-days old **(A, B)**, and 12–15-days old **(C, D)**. Flies were maintained in 12 h:12 h LD and then monitored for 5-days (3–5-days old) or 4-days (12–15-days old) in LD. Yellow represents the light period, and white represents the dark period of the 12 h:12 h LD cycle. Following LD, flies were monitored in constant darkness (free-running, white background) for at least 7 days. Morning anticipation phase score **(E)** of 3–5-day old (left) and 12–15-day old (right) control (white bars) and pathogenic (gray bars) flies maintained in 12 h:12 h LD and monitored for 4-days. Mean and SEM are shown. ****p < 0.0001, ns, not significant. Two-way ANOVA with genotype and age as factors, followed by Šídák's multiple comparisons test. *N* = 32 for 3–5-day old control and pathogenic flies, 49 for 12–15-day old control flies, and 40 for 12–15-day old pathogenic flies.

As locomotor data suggested altered activity and sleep patterns in flies modeling *C9orf72*-FTD, we were interested in observing circadian rhythm in these animals both in LD and during constant conditions. Double-plotted activity recordings (actograms) of control (left) and pathogenic (right) flies at 3–5-days old (top row) and 12–15-days old (bottom row) were generated using ClockLab and locomotor data from LD and constant dark were analyzed using Faas software (Figures 3A–D). Pathogenic flies of both ages were entrained in LD for 5 days but demonstrated reduced rhythmicity when released to constant darkness (free-running) (Table 1, Figures 3B, D). In LD, both young and old flies exhibited advanced morning anticipation (Figures 3B, D, E), indicated by reduced anticipation phase scores. When released to constant conditions (darkness), rhythmicity was reduced in pathogenic flies relative to controls at both ages (Table 1). In young flies, the proportion of rhythmic individuals was significantly lower than in controls (20/29 vs. 28/30; Fisher's exact test, *p* = 0.021). Similarly, in old flies, there was a significantly lower proportion of rhythmic individuals compared with controls (24/36 vs. 43/46; Fisher's exact test, *p* = 0.003). Robustness and power of rhythm were also analyzed. Robustness provides a measure of rhythm stability from cycle to cycle, and power of rhythm (Qp) indicates the strength and regularity of the rhythm. While both power and rubustness were reduced in pathogenic young and old flies, there was only a significant decrease in power of rhythm in 12–15-day-old flies modeling *C9orf72*-FTD (Table 1). Rhythmic pathogenic flies of both ages did not demonstrate significant changes in period length (Table 1). The reduced rhythmicity observed in flies modeling *C9orf72*-FTD was consistent with much older flies (5-6 weeks old) (Luo et al., 2012), *Clk* gene mutant flies (Allada et al., 1998), and flies modeling Alzheimer's Disease (Buhl et al., 2019).

**Table 1:** Circadian rhythm of flies under constant darkness conditions (DD, free running).

Genotype	Age (do)	Rhythmic	Arrhythmic	Rhythmic (%)	Period + SEM	Power + SEM	Robustness + SEM
*UAS-LDS-G4C2 (12)/y; elav-GAL4/+*	3–5	28	2	93.3	23.8 ± 0.12	65.1 ± 5.36	0.74 ± 0.05
*UAS-LDS-G4C2 (44)/ elav-GAL4*	3–5	20	9	^*^69.0	23.9 ± 0.08	40.6 ± 6.19	0.53 ± 0.07
*UAS-LDS-G4C2 (12)/y; elav-GAL4/+*	12–15	43	3	93.5	24.0 ± 0.19	85.4 ± 6.79	0.66 ± 0.04
*UAS-LDS-G4C2 (44)/ elav-GAL4*	12–15	24	12	^**^66.7	24.5 ± 0.10	^*^58.5 ± 7.81	0.53 ± 0.06

Pathogenic flies exhibited a lower percentage of rhythmic individuals than controls in both young and older age groups (*3–5 do*: 20/29 vs. 28/30, *p* = 0.021; *12–15 do*: 24/36 vs. 43/46, *p* = 0.003; Fisher's exact test). Period and rhythm robustness were not significantly different. Power of rhythm was significantly decreased in 12–15-day old pathogenic flies compared to 12–15-day old control flies. Arrhythmic flies were not included in period analysis but were included in power of rhythm and robustness analysis. ^*^p < 0.05, ^**^p < 0.01.

## 4 Discussion

Our data suggests that flies modeling *C9orf72*-FTD have increased nighttime activity and decreased nighttime sleep, which becomes more significant as they age. Older flies also displayed decreased sleep pressure during both day and night and reduced rhythmicity in free running. Intriguingly, young flies modeling *C9orf72*-FTD demonstrated altered day and night sleep latency, decreased sleep depth at night, and advanced morning anticipation in LD and reduced rhythmicity in free running. This suggests that changes in their circadian rhythm and sleep occur early in disease progression and provide an avenue for intervention.

Our findings contribute to a growing body of work that demonstrates disruption in sleep-wake cycles and circadian rhythm associated with neurodegeneration. These behaviors may be symptomatic of the degeneration of neuronal regions responsible for the regulation of sleep. However, increasing evidence suggests that disrupted sleep and circadian rhythm may have a more complicated relationship with neurodegeneration, with these behaviors contributing to processes that lead to disease, and thus serving as potential risk factors (Fifel and Videnovic, 2021). In flies modeling *C9orf72*-FTD, we observed fragmented sleep in young flies and fragmented and decreased total sleep as these flies aged. The lack of consolidated sleep, even when total sleep is not affected, has a deleterious effect on the glia-mediated mechanisms in flies that manage metabolic waste (Artiushin et al., 2018). This functions similarly to the glymphatic system in mammals, which is required to clear cellular debris in the brain, such as amyloid β and tau accumulation, and is a key contributor to neurodegeneration (Nedergaard and Goldman, 2020). Therefore, elucidating the molecular cause of these sleep/wake disruptions and decreased sleep pressure would have a significant impact on the management of neurodegenerative diseases.

Although these findings provide new insight, several limitations warrant consideration. First, this study relies on one specific hexanucleotide repeat expansion (HRE) model of FTD, and therefore studies one control condition (12 G4C2 repeats) and compares these animals to one pathogenic condition (44 G4C2 repeats). While this model has been studied previously to understand the molecular mechanisms of *C9orf72* pathogenicity (Goodman et al., 2019a,b), the toxic dipeptides produced are tagged with Green Fluorescent Protein (GFP), which while still resulting in toxic effects in flies (Goodman et al., 2019a,b), may not mimic the toxicity observed in human neurons and may actually underestimate the toxic effects (Morón-Oset et al., 2023). Secondly, these fly stocks have also been maintained in isolation and could harbor background mutations that may contribute to pathogenicity. Additionally, expression of these toxic dipeptides were induced using the enhancer of the neuronally expressed gene, *embryonic lethal abnormal vision* (*elav*). As the name implies, *elav* is expressed at all developmental stages, including early embryogenesis. While there are methods to limit expression to adult stages, we were concerned with additional variables that would be introduced, such as changing rearing temperature or exposure to exogenous compounds such as auxin, which has been shown to alter adult fly metabolism (Fleck et al., 2024). Lastly, only male flies were analyzed in this study. While this is not uncommon in *Drosophila* circadian studies because of mating status effects and egg-laying artifacts associated with female flies, it does not allow for investigation of differences in disease phenotype, progression, and severity linked to sex. To address these limitations, future studies should examine sleep, activity, and circadian rhythm in additional fly models that utilize varying expansions of G4C2 repeats (Balendra and Isaacs, 2018) in an untagged genetic context and control dipeptide expression using a gene enhancer specifically activated in adult neurons.

Sleep disturbances in patients with FTD often occur early in the disease and precede severe cognitive decline and behavior impairment (Anderson et al., 2009). This suggests that decreased sleep quality may act as an early diagnostic indicator, as well as a contributing factor to disease progression. Thus, intervention may be beneficial to the patient's quality of life. We observed similar findings in our young flies modeling *C9orf72*-FTD, with altered day and night sleep latency, decreased sleep depth at night, advanced anticipation, and reduced rhythmicity. Further studies investigating genetic modifiers of the observed sleep phenotypes, as well as external factors, such as light therapy, will contribute to our understanding of this disease phenotype. Because sleep is a highly conserved process, future findings should provide significant insight, as flies exhibit the fundamental characteristics of mammalian sleep, including catecholamine wake-promoting effects and regulation of major clock genes (Cirelli and Bushey, 2008; Dubowy and Sehgal, 2017). This suggests that molecular investigations of sleep–wake disturbances in *Drosophila* models of *C9orf72*-associated FTD may yield valuable knowledge for managing this disruptive aspect of the disease.

## Data Availability

The datasets presented in this study can be found in online repositories. The names of the repository/repositories and accession number(s) can be found below: https://doi.org/10.6084/m9.figshare.31343224.

## References

[B1] Akpoghiran O. Strich A. K. Koh K. (2025). Effects of sex, mating status, and genetic background on circadian behavior in Drosophila. Front. Neurosci. 18:1532868. doi: 10.3389/fnins.2024.1532868 39844849 PMC11750873

[B2] Allada R. White N. E. So W. V. Hall J. C. Rosbash M. (1998). A mutant Drosophila homolog of mammalian clock disrupts circadian rhythms and transcription of period and timeless. Cell 93, 791–804. doi: 10.1016/S0092-8674(00)81440-3 9630223

[B3] Anderson K. N. Hatfield C. Kipps C. Hastings M. Hodges J. R. (2009). Disrupted sleep and circadian patterns in frontotemporal dementia. Eur. J. Neurol. 16, 317–323. doi: 10.1111/j.1468-1331.2008.02414.x 19170747

[B4] Artiushin G. Zhang S. L. Tricoire H. Sehgal A. (2018). Endocytosis at the Drosophila blood-brain barrier as a function for sleep. Elife 7:e43326. doi: 10.7554/eLife.43326 30475209 PMC6255390

[B5] Balendra R. Isaacs A. M. (2018). *C9orf72*-mediated ALS and FTD: multiple pathways to disease. Nat. Rev. Neurol. 14, 544–558. doi: 10.1038/s41582-018-0047-2 30120348 PMC6417666

[B6] Buhl E. Higham J. P. Hodge J. J. L. (2019). Alzheimer's disease-associated tau alters Drosophila circadian activity, sleep and clock neuron electrophysiology. Neurobiol. Dis. 130:104507. doi: 10.1016/j.nbd.2019.104507 31207389

[B7] Cirelli C. Bushey D. (2008). Sleep and wakefulness in Drosophila melanogaster. Ann. N. Y. Acad. Sci. 1129, 323–329. doi: 10.1196/annals.1417.017 18591491 PMC2715168

[B8] Dedeene L. Van Schoor E. Vandenberghe R. Van Damme P. Poesen K. Rudolf Thal D. (2019). Circadian sleep/wake-associated cells show dipeptide repeat protein aggregates in *C9orf72*-related ALS and FTLD cases. Acta Neuropathol. Commun. 7:189. doi: 10.1186/s40478-019-0845-9 31791419 PMC6889626

[B9] DeJesus-Hernandez M. Mackenzie I. R. Boeve B. F. Boxer A. L. Baker M. Rutherford N. J. . (2011). Expanded GGGGCC hexanucleotide repeat in noncoding region of *C9ORF72 c*auses chromosome 9p-linked FTD and ALS. Neuron 72, 245–256. doi: 10.1016/j.neuron.2011.09.011. 21944778 PMC3202986

[B10] Dubowy C. Sehgal A. (2017). Circadian rhythms and sleep in Drosophila melanogaster. Genetics 205, 1373–1397. doi: 10.1534/genetics.115.185157 28360128 PMC5378101

[B11] Farágo A. Zsindely N. Bodai L. (2019). Mutant huntingtin disturbs circadian clock gene expression and sleep patterns in Drosophila. Sci. Rep. 9:7174. doi: 10.1038/s41598-019-43612-w 31073199 PMC6509128

[B12] Fifel K. Videnovic A. (2021). Circadian and sleep dysfunctions in neurodegenerative disorders-an update. Front. Neurosci. 14:627330. doi: 10.3389/fnins.2020.627330 33536872 PMC7848154

[B13] Fleck S. A. Biswas P. DeWitt E. D. Knuteson R. L. Eisman R. C. Nemkov T. . (2024). Auxin exposure disrupts feeding behavior and fatty acid metabolism in adult Drosophila. Elife 12:RP91953. doi: 10.7554/eLife.91953 38240746 PMC10945601

[B14] Giebultowicz J. M. Long D. M. (2015). Ageing and circadian rhythms. Curr. Opin. Insect Sci. 7, 82–86. doi: 10.1016/j.cois.2015.03.001 26000238 PMC4435573

[B15] Goodman L. D. Prudencio M. Kramer N. J. Martinez-Ramirez L. F. Srinivasan A. R. Lan M. . (2019b). Toxic expanded GGGGCC repeat transcription is mediated by the PAF1 complex in *C9orf72*-associated FTD. Nat. Neurosci. 22:863. doi: 10.1038/s41593-019-0396-1 31110321 PMC6535128

[B16] Goodman L. D. Prudencio M. Srinivasan A. R. Rifai O. M. Lee V. M. Petrucelli L. . (2019a). eIF4B and eIF4H mediate GR production from expanded G4C2 in a Drosophila model for *C9orf72*-associated ALS. Acta Neuropathol. Commun. 7, 62–69. doi: 10.1186/s40478-019-0711-9 31023341 PMC6485101

[B17] Harrisingh M. C. Wu Y. Lnenicka G. A. Nitabach M. N. (2007). Intracellular Ca2+ regulates free-running circadian clock oscillation *in vivo*. J. Neurosci. 27, 12489–12499. doi: 10.1523/JNEUROSCI.3680-07.2007 18003827 PMC6673328

[B18] Hendricks J. C. Finn S. M. Panckeri K. A. Chavkin J. Williams J. A. Sehgal A. (2000). Rest in Drosophila is a sleep-like state. Neuron 25, 129–138. doi: 10.1016/S0896-6273(00)80877-6 10707978

[B19] Larsen E. Della Monica C. Hassanin H. Atzori G. Dijk D. Revell V. L. . (2023). Sleep disturbance associated with temporal lobe degeneration in frontotemporal dementia. Alzheimer's Dement. 19:e073890. doi: 10.1002/alz.073890

[B20] Linford N. J. Chan T. P. Pletcher S. D. (2012). Re-patterning sleep architecture in Drosophila through gustatory perception and nutritional quality. PLoS Genet. 8:e1002668. doi: 10.1371/journal.pgen.1002668 22570630 PMC3342939

[B21] Luo W. Chen W. Zhifeng Y. Chen D. Sowcik M. Sehgal A. (2012). Old flies have a robust central oscillator but weaker behavioral rhythms that can be improved by genetic and environmental manipulations. Aging Cell 11, 428–438. doi: 10.1111/j.1474-9726.2012.00800.x 22268765 PMC3353743

[B22] Mizielinska S. Grönke S. Niccoli T. Ridler C. E. Clayton E. L. Devoy A. . (2014). *C9orf72* repeat expansions cause neurodegeneration in Drosophila through arginine-rich proteins. Science 345, 1192–1194. doi: 10.1126/science.1256800 25103406 PMC4944841

[B23] Morón-Oset J. Fischer L. K. Carcole M. Giblin A. Zhang P. Isaacs A. M. . (2023). Toxicity of *C9orf72*-associated dipeptide repeat peptides is modified by commonly used protein tags. Life. Sci. Alliance 6:e202201739. doi: 10.26508/lsa.202201739 37308278 PMC10262077

[B24] Namgyal D. Lim C. (2025). Circadian rhythm dysfunction in neurodegenerative diseases: a bidirectional perspective and therapeutic potential. Nat. Sci. Sleep 17, 2969–2989. doi: 10.2147/NSS.S561326 41287625 PMC12640578

[B25] Nedergaard M. Goldman S. A. (2020). Glymphatic failure as a final common pathway to dementia. Science 370, 50–56. doi: 10.1126/science.abb8739 33004510 PMC8186542

[B26] Renton A. E. Majounie E. Waite A. Simon-Sanchez J. Rollinson S. Gibbs J. R. (2011). A hexanucleotide repeat expansion in *C9ORF72* is the cause of chromosome 9p21-linked ALS-FTD. Neuron 72, 257–268. doi: 10.1016/j.neuron.2011.09.010. 21944779 PMC3200438

[B27] Shaw P. J. Cirelli C. Greenspan R. J. Tononi G. (2000). Correlates of sleep and waking in Drosophila melanogaster, Science 287, 1834–1837. doi: 10.1126/science.287.5459.1834 10710313

[B28] Sokolove P. G. Bushell W. N. (1978). The chi square periodogram: its utility for analysis of circadian rhythms. J. Theor. Biol. 72, 131–160. doi: 10.1016/0022-5193(78)90022-X 566361

[B29] Tataroglu O. Emery P. (2014). Studying circadian rhythms in Drosophila melanogaster. Methods 68, 140–150. doi: 10.1016/j.ymeth.2014.01.001 24412370 PMC4049855

[B30] Vecsey C. G. Koochagian C. Porter M. T. Roman G. Sitaraman D. (2024). Analysis of sleep and circadian rhythms from Drosophila activity-monitoring data using SCAMP. Cold Spring Harb. Protoc. 2024:pdb.prot108182. doi: 10.1101/pdb.prot108182 38336392 PMC11552080

[B31] Walker N. Vaughn B. (2021). 803 sleep disturbances in patients with frontotemporal dementia. Sleep 44, A312–A314. doi: 10.1093/sleep/zsab072.800

[B32] Wiggin T. D. Goodwin P. R. Donelson N. C. Liu C. Trinh K. Sanyal S. . (2020). Covert sleep-related biological processes are revealed by probabilistic analysis in Drosophila. Proc. Natl. Acad. Sci. USA. 117, 10024–10034. doi: 10.1073/pnas.1917573117 32303656 PMC7211995

[B33] Ziehm M. Piper M. D. Thornton J. M. (2013). Analysing variation in Drosophila aging across independent experimental studies: a meta-analysis of survival data. Aging Cell. 12, 917–922. doi: 10.1111/acel.12123 23795998 PMC3963443

[B34] Zu T. Pattamatta A. Ranum L. P. W. (2018). Repeat-associated non-ATG translation in neurological diseases. Cold Spring Harb. Perspect. Biol. 10: a033019. doi: 10.1101/cshperspect.a033019 29891563 PMC6280704

